# Monitoring mandibular movements to detect Cheyne-Stokes Breathing

**DOI:** 10.1186/s12931-017-0551-8

**Published:** 2017-04-20

**Authors:** Jean-Benoît Martinot, Jean-Christian Borel, Nhat-Nam Le-Dong, Hervé Jean-Pierre Guénard, Valerie Cuthbert, Philip E. Silkoff, David Gozal, Jean-Louis Pepin

**Affiliations:** 1 Centre du Sommeil et de la Vigilance, CHU UCL Namur Site Ste Elisabeth, 15, Place Louise Godin, 5000 Namur, Belgium; 2 AGIR à dom. Association, 38240 Meylan, France; 3 RespiSom, Chaussée de Marche 571, 5101 Erpent, Belgium; 4 Laboratory of Physiology, Bordeaux University, Bordeaux, France; 5 Temple University, Philadelphia, PA USA; 6 University of Chicago, Chicago, IL USA; 7 University Grenoble Alpes, HP2 INSERM U1042, 38000 Grenoble, France; 8 CHU de Grenoble, Laboratoire EFCR, Pôle THORAX et VAISSEAUX, Grenoble, France

**Keywords:** Central sleep apnea syndrome, Cheyne Stokes breathing, Sleep mandibular movements, Polysomnography

## Abstract

**Background:**

The patterns of mandibular movements (MM) during sleep can be used to identify increased respiratory effort periodic large-amplitude MM (LPM), and cortical arousals associated with “sharp” large-amplitude MM (SPM). We hypothesized that Cheyne Stokes breathing (CSB) may be identified by periodic abnormal MM patterns. The present study aims to evaluate prospectively the concordance between CSB detected by periodic MM and polysomnography (PSG) as gold-standard.

The present study aims to evaluate prospectively the concordance between CSB detected by periodic MM and polysomnography (PSG) as gold-standard.

**Methods:**

In 573 consecutive patients attending an in-laboratory PSG for suspected sleep disordered breathing (SDB), MM signals were acquired using magnetometry and scored manually while blinded from the PSG signal. Data analysis aimed to verify the concordance between the CSB identified by PSG and the presence of LPM or SPM. The data were randomly divided into training and validation sets (985 5-min segments/set) and concordance was evaluated using 2 classification models.

**Results:**

In PSG, 22 patients (mean age ± SD: 65.9 ± 15.0 with a sex ratio M/F of 17/5) had CSB (mean central apnea hourly indice ± SD: 17.5 ± 6.2) from a total of 573 patients with suspected SDB. When tested on independent subset, the classification of CSB based on LPM and SPM is highly accurate (Balanced-accuracy = 0.922, sensitivity = 0.922, specificity = 0.921 and error-rate = 0.078). Logistic models based odds-ratios for CSB in presence of SPM or LPM were 172.43 (95% CI: 88.23–365.04; *p* < 0.001) and 186.79 (95% CI: 100.48–379.93; *p* < 0.001), respectively.

**Conclusion:**

CSB in patients with sleep disordered breathing could be accurately identified by a simple magnetometer device recording mandibular movements.

## Summary at a glance

Cheyne Stokes Breathing (CSB) is a poor prognosis sleep condition that should be screened for in patients with heart failure or other severe neurological or kidney diseases. A simple device that detects mandibular movements can accurately identify patients with CSB.

## Background

Central apnea/hypopnea assuming the pattern of Cheyne Stokes breathing (CSB) is an independent risk condition for mortality in patients with chronic heart failure with a prevalence similar to obstructive sleep apnea or hypopnea (OSA) reaching 30% [1–3]. CSB has also been described in patients who have experienced cerebrovascular accidents and among those with end-stage renal disease [4]. In addition, the presence of idiopathic central apneas carries an increased risk of atrial fibrillation [5]. Thus, screening and early detection of CSB could be valuable in large populations of chronic disease at-risk patients.

CSB is a particular form of periodic waxing and waning respiration. The diagnosis is made in presence of periods of central apnea or hypopnea alternating with a crescendo/decrescendo pattern of ventilation. The International Classification of Sleep Diseases diagnostic criteria for CSB requires 10 central apneas per hour of sleep. The cycle length (the time from one zenith in airflow during the respiratory phase to the next zenith in airflow) varies with the underlying disease. In systolic heart failure, cycle lengths are longer (between 45 and 90 s), when compared to the cycle lengths reported for other disorders associated with CSB (~35 s) [6]. During the crescendo/decrescendo phases, respiratory efforts are driving changes in ventilation reflecting variations in respiratory drive. Recurrent arousals occur usually at ventilation peaks, promote ventilatory instability, and perpetuate CSB breathing patterns [4, 7]. The diagnosis requires expensive and poorly accessible type 1 or 2 polysomnography but could be addressed with a portable monitoring in a high pre-test probability population of CSB for early detection and intervention.

We have previously showed that respiratory effort are well characterized by an increase in the amplitude of mandibular movements (MM) > 0.3 mm during episodes of OSA [8, 9]. In addition, we have also documented that MM reliably identify cortical arousals ending respiratory events and closing the mouth [10, 11]. The MM patterns observed are 1) large-amplitude MM (MML), and 2) “sharp” large-amplitude MM (MMS), and these two specific and easily recognizable features readily identify respiratory efforts and cortical arousals, respectively [11]. Because MM are simple to acquire using magnetometry, and since the quality of MM signals remains more stable than nasal flow, throughout the night, measurements of MM could be used as a single channel monitor or screener, particularly for use in out of sleep laboratory conditions use [11].

Based on early empirical observations of magnetometry recordings during CSB events, we hypothesized that periodic MML (LPM) and periodic MMS (SPM) would identify classical CSB which is characterized by periods of respiratory effort with increased ventilation alternating with periods of no (or substantially reduced) effort during apneas (or hypopneas). We therefore evaluated the concordance of LPM and SPM with CSB identified during in-lab PSG.

## Methods

### Study design

This prospective study included all adults patients referred to a sleep laboratory (University Hospital UCL Namur site St Elisabeth, Belgium) over a 12-month period for in-laboratory PSG for evaluation of suspected sleep disordered breathing (SDB) of moderate to high probability. The study met the standards of the Declaration of Helsinki, was approved by the Medical Ethics Committee of the Clinique et Maternité Sainte Elisabeth Namur Belgium (approval #B166201215073), and all participants provided written informed consent prior to study commencement.

PSG scoring was performed by trained technicians strictly following the American Academy of Sleep Medicine (AASM) rules [6]. To maximize the visibility of respiratory events of interest, the total sleep period was divided into 3-min segments, and typical events of CSB were identified. CSB periods consisted of at least five breaths demonstrating waxing and waning flow amplitude and separated by central apnea or hypopnea. The duration of CSB periods was analyzed to measure the cycle length.

### Analysis of mandibular movements (MM) signals (Fig. 1)

**Fig. 1:**
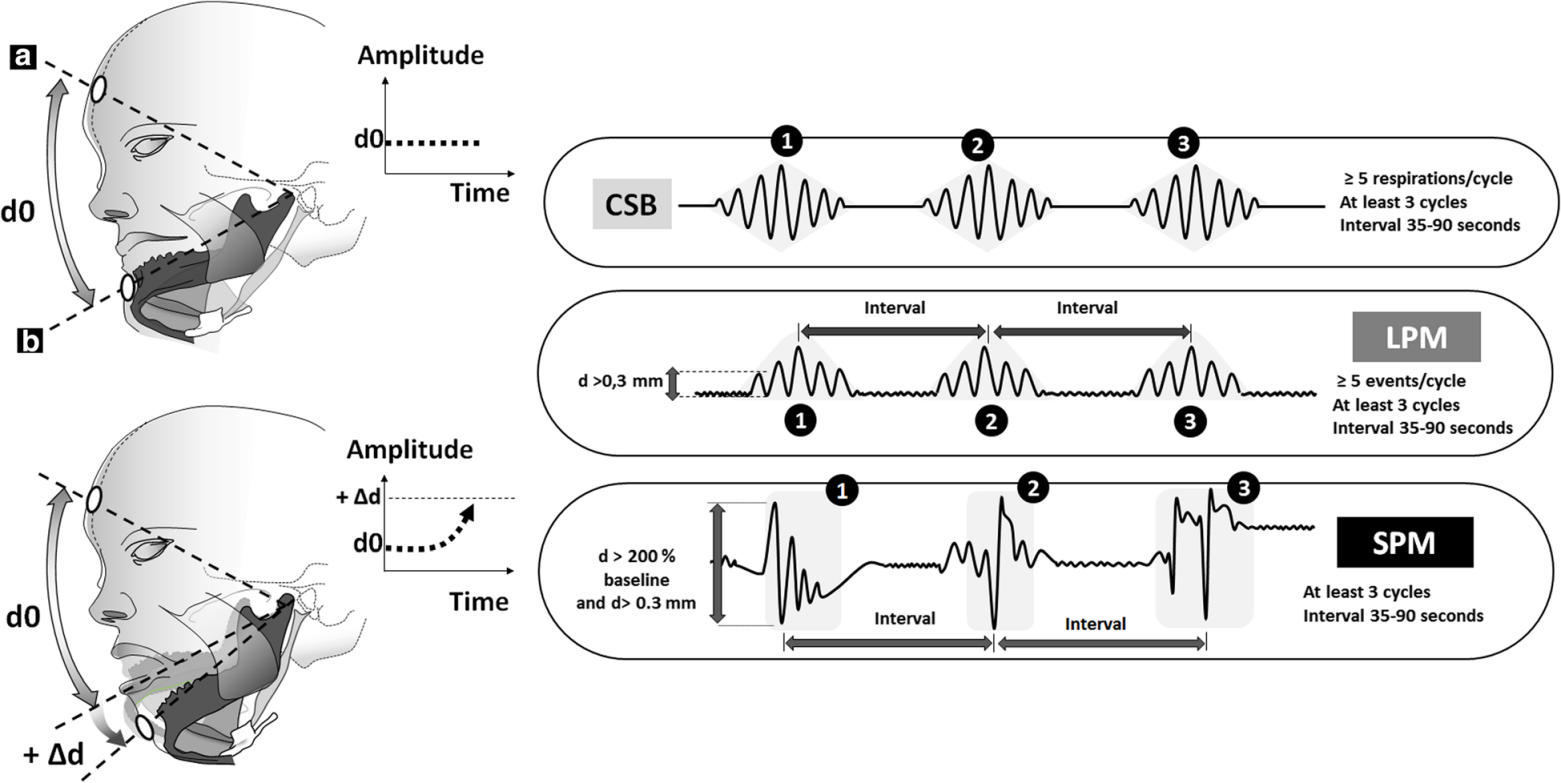
Measuring the mandibular movements and definition of periodic MML and MMS. **a** Forehead sensor, **b** Chin emitter, d is a distance between emitter and sensor, d0: offset level, Δd: variation of distance d when the mouth opens. CSB: Cheyne-Stokes breathing highlighted by a flow typical crescendo –decrescendo pattern of at least 5 respirations; SPM: periodic sharp mandibular movements occurring on cortical arousals during the hyperventilation phase (these are unevenly observed); LPM: periodic large mandibular movements accompanying the changes in flow during the hyperventilation phase

The MM signal was acquired during the PSG. However, scoring was performed manually by two scorers blinded from the rest of the PSG signals. Prior to main analysis, the inter-observer agreement was evaluated for both SPM and LPM in 100 different samples, each one contains 200 randomised observations. The result shows a high agreement level between two scorers with Cohen’s kappa coefficients of 0.89 ± 0.03 (min = 0.82, max = 0.95) and 0.88 ± 0.03 (min = 0.80, max = 0.93) for SPM and LPM, respectively. The within-observer was also evaluated by validating the manual classification of periodic MM against Central respiratory event. Such validation was also repeated through 100 different samples of 200 randomised observations. The Cohen’s Kappa coefficients were steadily above 0.81. Mean Kappa coefficient for both scorers was 0.88 ± 0.03 (min = 0.81, max = 0.94).

Normal MM signals consist of oscillatory displacements of the mandible with inspiration and expiration with an amplitude ≤ 0.3 mm (Fig. 1). MM > 0.3 mm have been shown to occur during increased respiratory effort and are denominated as large MM (MML); these displacements occur at the breathing frequency. Sharp large-amplitude MM occur during cortical arousals, are > 3 mm, and termed as MMS. These movements participate to mouth closure. They are all the time well depicted because greater than 200% with regards to the baseline mandibular motion [12]. Arbitrarily, periodic MM had to be present at least three times during the 3-min fragments to be considered as a period of CSB. The periodic occurrence of MM was searched by examining the time period between LPM and SPM as shown in Fig. 1.

### Study measurements

Routine laboratory-based PSG were recorded with B3iP® Medatec Brussel Belgium. The parameters monitored included EEG (Fz-A+, Cz-A+, Pz-A+), right and left electro-oculogram, submental EMG, tibial EMG, chest and abdominal wall motion by respiratory inductance plethysmography (SleepSense S.L.P.Inc, St Charles, USA), nasal and oral flows respectively with a pressure transducer and a thermistor, and O2 saturation by digital oximeter displaying pulse waveform (Nonin®, Nonin Medical, Plymouth USA) [13].

**Fig. 2:**
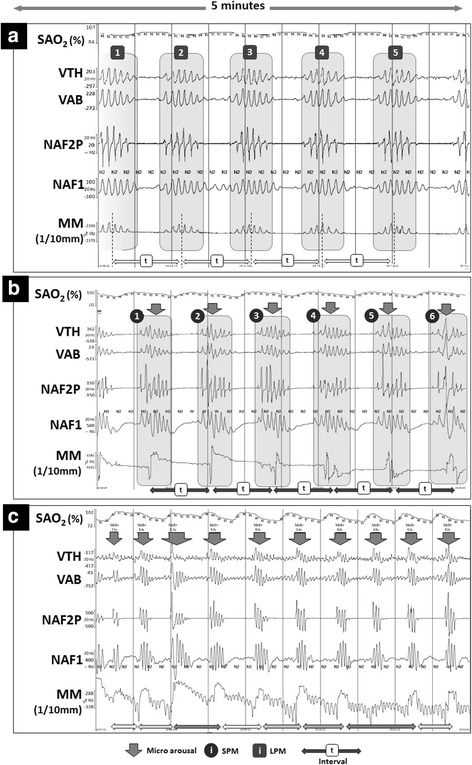
Typical MM recorded during CSB vs Obstructive events for comparing true positive SPM or LPM vs MM during obstructive events. Examples of: **a**, **b** Central and **c** Obstructive respiratory events during a period of 5 sleep minutes. The arrows indicate cortical arousals. SaO2: oxygen saturation; VTH and VAB: thoracic and abdominal inductance belts; NAF2P and NAF1: nasal pressure transducer and oronasal thermal flow sensor; MM: mandibular movements. Cortical arousals are highlighted with an arrow. During the central event (**a**, **b**), one issue of classification is presented, including: True Positive for LPM (**a**) and for SPM (**b**). During the obstructive event (**c**), the more negative the signal, the lower the mandibular position and the greater the mouth opening until a sharp and great movement occurs closing the mouth

A mid-sagittal MM magnetic sensor (Brizzy® Nomics, Liege, Belgium) measured the distance in mm between two parallel, coupled, resonant circuits placed on the forehead and on the chin (Fig. 1). The transmitter generates a pulsed magnetic wave of low energy, at 10Hz. The change in the magnetic field is inversely related to the cube of the distance (d) between the chin and forehead probes. The probes were connected to an electronic module, and the distance was computed with a resolution of 0.1 mm before transmission to the PSG through a wireless connection. For each measurement, the zero value of the mandibular displacement was assigned to a position of completely closed mouth. The signal was processed in such a way that, when the distance between the probes increases, it actually decreases. Therefore, the more negative the signal, the lower the mandibular position and the greater the mouth opening [8]. Mandibular-movement variables are described in Table 1, and depicted in Figs. 1, and 2.

**Table 1:** Frames of mandibular-derived variables

MM Variable	Symbol	Categorization
At least 5 respiratory cycles during which MM amplitudes are ≥ 0.3 mm is termed a period of periodic large mandibular movements and follows the typical crescendo-decrescendo pattern of CSB.	LPM	Present or absent
At least one sharp and large MM (amplitude > 3 mm) during the hyperventilation phase or during a period of apnea. These are associated with a cortical arousal.	SPM	Present or absent

Note: A SPM results in mouth closure (on cortical arousal) whether occurring with or without a LPM. A SPM can occur during the hyperventilation phase or during the apneic period. During the hyperventilation phase, SPM is disruptive the breathing frequency.

### Statistical methods

LPM and SPM (Table 1) were handled as binary factor (present or absent) after the lifting of the blind reading procedure.

To verify whether CSB could be correctly classified using LPM/SPM, two classification algorithms were used:

1. A logistic model [14] consisting of a linear regression algorithm that predicts the probabilities of a binary dependent variable (CSB) as a function of two independent variables (SPM and LPM). This model also allows the evaluation of the relationship between the outcome and the LPM or SPM by estimating odds-ratios (OR).
2. Classification and Regression Trees (CART), which is a non-parametric classification method introduced in 1984 by Breiman et al. [15]. This algorithm constructs a model by recursively partitioning the source data into smaller subsets and fitting a simple rule within each partition. As the result, the final model can be graphically presented as a binary tree on which the leaves represent the class of target variable (CSB: negative or positive), two nodes represent the predictors (SPM and LPM), and several branches represent binary conjunction rules for each predictor.

**Fig. 3:**
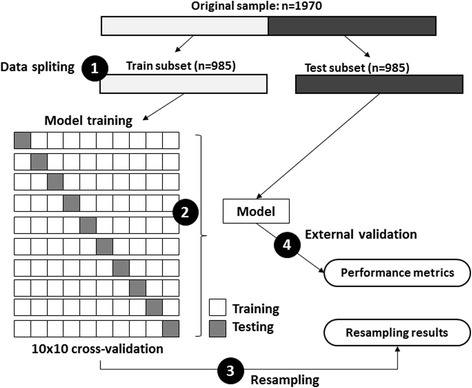
Model training and testing process. (*1*) data splitting: the original dataset (*n* = 1970) were randomly divided into two equal sized subsets of fragments (*n* = 985): one to be used for model training and the other for model testing. (*2*) the model training process implies a 10 × 10 cross-validation and provides the best fit model. (*3*) Posterior predictive values of the model were estimated from cross-validation resampling process. (*4*) Finally, the model was tested against external subset (*n* = 985). Model’s performance metrics were evaluated

The original dataset (1970 5-min segments) was randomly divided into two equal parts: one was used for model training (985 5-min segments) and the other for validation (985 5-min segments). The model training involved a 10 × 10 cross-validation, in which the original training dataset is randomly partitioned into 10 equal sized blocks. Nine blocks were used as training data while one remaining block was retained for testing the model. This process was repeated ten times so each of the ten blocks would be used exactly once as for the validation data. Those 100 results were averaged to obtain the final model with the best accuracy. This final model has been tested again over a large external validation subset to ensure its generalized value. The performance of models was evaluated by 10 metrics (Fig. 3).

All data analysis was conducted in R programming language (Foundation for Statistical Computing, Vienna, Austria) [16]. Model training and external validation were performed using *mlr* package (https://cran.r-project.org/web/packages/mlr/index.html). Following packages were required for building the classifiers: *stats* (logistic model) and *rpart* (CART model) [17].

## Results

A group of 22 patients was identified with CSB in 573 consecutive recorded subjects; in this group, a total of 1,970 3-min sleep segments were evaluated (CSB was confirmed in 1,060 fragments). Patient characteristics are summarized in Table 2. Twelve patients had congestive heart failure (mean left ventricular ejection fraction < 30%), five patients has sustained a cerebrovascular accident (CVA), and in five patients, the etiology of CSB was unknown. No patient was on opioids. Compared to the original dataset, the training set of 985 5-min fragments had the same distribution pattern of CSB, LPM and SPM.

**Table 2:** Population characteristics of 22 patients being evaluated in the sleep laboratory for suspected sleep-disordered breathing

	Median	Mean ± SD	95% CI
Age (yrs)	67	65.95 ± 15.02	51.95–85.47
Height (cm)	169	169.86 ± 6.84	167.29–172.43
Weight(kg)	83	87.81 ± 13.04	73.0–112.2
BMI (kg/m2)	29.35	30.52 ± 4.91	24.78–41.22
LVEF (%)^a^	30	33.33 ± 9.85	28.22–38.44
TST (min)	448	441.52 ± 95.14	321.2–521.3
AHI (n/h)	57.5	52.93 ± 21.23	18.86–83.63
CAI (n/h)	19.2	17.50 ± 6.20	5.70–38.40
Arousal index (n/h)	42.3	40.67 ± 17.92	15.5–68.34

Note: ^a^LVEF: Left ventricular ejection fraction, only measured in the group of HF (*n* = 12) patients. TST: total sleep time, AHI: Apnea-Hypopnea hourly index, CAI: central apnea hourly indice. Sex: F = 5 (22.7%), M = 17 (77.3%)

### Results obtained with the models training to classify fragments with mandibular movements

**Fig. 4:**
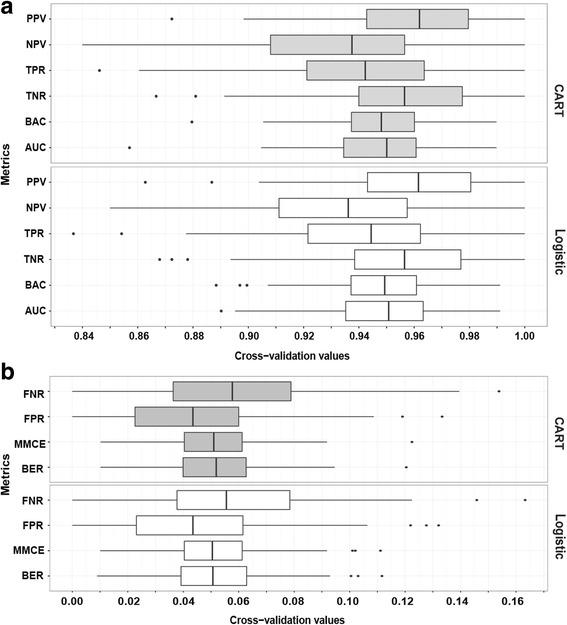
Performance of the two models (CART and Logistic) evaluated by cross-validation resampling. **a** Six metrics for evaluating the model’s performance (Best value = 1): PPV = Positive predictive value or the probability that fragments with positive LPM or SPM truly reflect CSB. NPV = Negative predictive value or the probability that CSB is correctly excluded once neither LPM nor SPM is identified; TPR = True positive rate, or Sensitivity, is the percentage of correctly classified observation among positive CSB class; TNR = True Negative rate or Specificity, is the percentage of correctly excluded CSBs; BAC = Balanced accuracy or Mean of true positive rate and true negative rate; AUC = Area under Receiver Operating Curve (ROC) that results from computing False positive rate and True positive rate from many thresholds. **b** Four metrics for evaluating the classification error (Best value =0): FNR = False negative rate, or percentage of in the negative CSB class. FPR = False positive rate, or percentage of misclassified observations in the Positive CSB class, MMCE = mean misclassification error, defined as mean of all classifications that disagree with truth; BER = balanced error index, defined as Mean of misclassification error rates on all individual classes

The performance of two models used for classification during training on 985 5-min segments is presented in Fig. 4. The validation of the trained classifiers is shown in Tables 3 and 4. The classification outcome is presented as a confusion matrix (Table 3) and was identical for both models after testing. The classification of CSB based on LPM and SPM was accurate in 92.2%. Each classifier provided excellent sensitivity and specificity: 92.3% and 92.1%, respectively (Table 4).

**Table 3:** Confusion matrix obtained during the testing procedure

	Classification by model		
Real observed	Positive	Negative	Total
Positive	TP = 489	FN = 41	530
Negative	FP = 36	TN = 419	455
Total	525	460	985

Note: The result was identical for both models

The Cohen’s Kappa coefficient is 0.84 (95% CI: 0.81–0.88, *p* < 0.001)

*TP* true positive, *FP* false positive, *FN* false negative, *TN* true negative

**Table 4:** Performance of the two models, evaluated on an external dataset

Metrics	Scale (Worst-Best)	Logistic model	Decision tree model
ROC-AUC	0.5–1	0.93	0.92
Balanced Accuracy	0–1	0.92	0.92
True positive rate (Sensitivity)	0–1	0.92	0.92
True negative rate (Specificity)	0–1	0.92	0.92
Positive predictive value	0–1	0.93	0.93
Negative predictive value	0–1	0.91	0.91
False negative rate	1-0	0.08	0.08
False positive rate	1-0	0.08	0.08
Balance error	1-0	0.08	0.08
Mean misclassification error index	1-0	0.08	0.08

### Logistic model and odds ratio for CSB-PSG

To optimally estimate the statistical power of LPM and SPM in identification of PSG-CSB, we selected a traditional logistic model (Table 5). The logistic model showed that MML or MMS contributes independently and significantly to the classification of CSB. The odds ratio for CSB increases 172 folds (95% CI: 88.23–365.04) and 186 folds (95% CI: 100.48–379.93) when SPM or LPM are present, respectively.

**Table 5:** Simple logistic model

Simple logistic	Coefficient (97.5% CI)	Odds-ratio (97.5% CI)	*p*-value
Intercept	−2.54 (−2.91– − 2.21)	0.08 (0.05–0.11)	<0.00001
LPM (+)	5.23 (4.61–5.94)	186.79 (100.48–379.93)	< 0.00001
SPM (+)	5.15 (4.48–5.90)	172.43 (88.23–365.04)	< 0.00001

AIC (Akaike Information Criterion) = 426.16

### Decision tree classifier (CART)

**Fig. 5:**
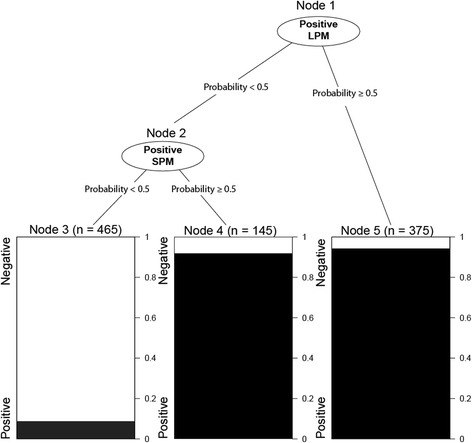
Decision tree classifier for detecting CSB by periodic MML and MMS. Decision tree classification rule: First, the model checks whether LPM presents within the segment (*Node 1*). If so, the segment will be classified as CSB (*Node 5*). If not, the model will check whether the segment presents a SPM (*Node 2*). If so, it will be classified as CSB positive (*Node 4*); if not, CSB will be excluded (*Node 3*). Based on such rule, absence of both LPM and SPM allows to exclude CSB with an error rate of 8.6% whilst CSB could be ruled in by using either LPM (error rate of 5.9%) or SPM (error rate of 8.3%)

The decision tree classifier is most suitable for daily use in clinical settings, as it provides a straightforward decision rule and require no calculation. Based on this classifier, presence of both LPM or SPM correctly identify CSB with an error rate of 5.9%; in contrast, absence of both LPM or SPM excluded CSB with an error rate of 8.6% (Fig. 5).

## Discussion

In the present study, scoring of the MM signals reliably indicated the presence or absence of CSB in patients being evaluated for suspected SDB. Our findings show that the presence of LPM and/or SPM can accurately detect CSB because the periodicity of these typical movements of the mandible. In contrast, MM ruled out CSB in absence of LPM and SPM. These findings support conducting a prospective validity assessment studies of MM recordings in a home-based type 4 device to detect CSB in chronic disease at-risk populations.

Use of MM signals has emerged as sensitive and specific for identifying underlying respiratory events during OSA [9]. Furthermore, sharp large-amplitude movements of the mandible (MMS) detect cortical arousals that occur after OSA events [12]. In the presence of respiratory efforts, even without pharyngeal obstruction, the forces produced by the displacement of the thorax are transmitted through the mediastinum to the upper airway. During each breath, the negative swings of intrathoracic pressure exert caudal traction stretching and improving upper airway patency [18]. Accordingly, similar to the MM patterns observed in OSA, changes in mandibular position directly reflect changes in respiratory effort during the hyperventilation phase of CSB. During the apneic period, no MM is observed.

In the present study, classification approaches using two binary predictors (LPM and SPM) reliably detected a single binary response variable (presence of CSB detected by PSG). Although different statistical methods may differ in their algorithms, their level of complexity and their overall performance, decision rules in classifying and analyzing the relationship between the binary predictors (LPM, SPM) and the predicted in-laboratory PSG CSB were clearly apparent and involved interpretable models (a traditional logistic model and a decision tree (CART model). Compared to a traditional logistic model and odds-ratio, the CART algorithm has more advantages as the decision tree can be visualized and easily interpreted by straightforward prediction rules, and accordingly is simple and robust for clinical decision making [17]. As the validation results were identical for both logistic and CART models, any of them could be used to predict the probability of CSB using MM signals.

Notwithstanding the unique predictability of CSB from MM recordings, we should mention some potential limitations of MM signals. MM signals may fade or become less easily detectable in presence of the muscle atonia during REM sleep. However, CSB is more common during NREM sleep and usually abolished during REM sleep [19, 20].

MM signal artefacts from magnetic interferences have only been observed in CT scan rooms or in presence of metal parts placed very close to the probes, or moving in the space around or between the probes. Steady state magnetic signals are filtered out [21].

Some drugs such as sedatives could increase the threshold for arousals and accordingly reduce the frequency of arousal events, and therefore of SPM, affecting the ability of the signal to detect CSB [22]. This risk is limited in a model where two independent variables (LPM and/or SPM) are employed. Others movements of the mandible (due to bruxism, chewing or swallowing) have been described during sleep but the latter are void of the typical CSB periodicity. They are not driven at the breathing frequency [19]. The false negative and positive rates could have been affected by scoring error and the simplicity of the classification rule (exclusive use of binary predictor). However, the study is strengthened by the blinded scoring approach, the large sample size used with random assignments into a training and validation sets, a large and comprehensive range of classifiers with potential machine solutions, a repeated K-fold cross validation on random data subsets, and an independent and random model of validation. Based on the present findings, we posit that the models developed herein could contribute the development of an algorithm to evaluate CSB diagnosis based on MM manual analysis, particularly considering that the CART model classification tree is appropriate for human decision making [23]. Of note, even more complex algorithms may have potential applications when used in the development of automated interpretation software.

## Conclusion

Isolated analysis of mandibular movements enables accurate identification of CSB in patients during in lab PSG. MM may be a useful simple tool for screening and home monitoring of patients at risk for CSB.

## Abbreviations

CART: Classification and regression tree
CSB: Cheyne stokes breathing
LPM: Large periodic mandibular movement
MM: Mandibular movements
MML: Period of at least 5 respiratory cycles during which peak to peak Mandibular Movement is of at least 0.3 mm and terminating with an arousal
MMS: Sharp and sudden Movement of the Mandible of at least 3 mm and twice above the average of the previous mandibular movements
OSA: Obstructive sleep apnea
PSG: Polysomnography
SDB: Sleep disordered breathing
SPM: Sharp periodic mandibular movement
